# Effect of vegetation on cutaneous leishmaniasis in Paraná, Brazil

**DOI:** 10.1590/0074-02760170505

**Published:** 2018-07-05

**Authors:** Helen Aline Melo, Diogo Francisco Rossoni, Ueslei Teodoro

**Affiliations:** 1 Universidade Estadual de Maringá, Programa de Pós-Graduação em Ciências da Saúde, Maringá, PR, Brasil; 2 Universidade Estadual de Maringá, Departamento de Estatística, Maringá, PR, Brasil

**Keywords:** cutaneous leishmaniasis, zoonoses, environmental health, spatial analysis, spatial regression

## Abstract

**BACKGROUND:**

Cutaneous leishmaniasis (CL) is endemic in the state of Paraná, Brazil.

**OBJECTIVE:**

This study aimed at analysing the influence of the remaining native vegetation on the prevalence of CL in Paraná.

**METHODS:**

Global testing was used for spatial autocorrelation along with simultaneous autoregressive model (SAR). The regression was based on the CL coefficient (cases/100,000 inhabitants) as a function of the percentage of natural vegetation cover, altitude, total number of cases, and spatial density (SD) per km^2^; the location data of the Paraná state municipalities and the detection coefficient (DC) (cases/100,000 inhabitants) of autochthonous cases of CL were obtained from the SINAN in 2012 and 2016. Data on the remaining forests were collected from the Fundação SOS Mata Atlântica and Instituto Nacional de Pesquisas Espaciais.

**FINDINGS:**

The spatial regression of DC revealed statistical significance for SD (Z = 24.1359, p < 0.05, 2012-2013; Z = 24.0817, p < 0.05, 2013-2014; Z = 33.4824, p < 0.05, 2014-2015; and Z = 27.1515, p < 0.05, 2015-2016.

**CONCLUSIONS:**

CL cases are reported in areas with native vegetation, such as in riparian forests. However, vegetation is not the only variable that influences the incidence of CL.

The prevalence of cutaneous leishmaniasis (CL) is reported in 97 countries, including America and those in Europe, Africa, and Asia, with 0.7-1.3 million new cases registered annually (WHO 2017). This disease is one of the six most severe infectious diseases worldwide due to its high detection coefficient (DC) and capability to cause deformations in patients (MS 2016). In Brazil, from 1993 to 2016, the annual average number of CL cases was 22,140, with a DC of 9.8 cases/100,000 inhabitants (MS 2016). CL is endemic in Brazil. In the early 1980s, autochthon cases were reported in 19 states. Moreover, in 2003, there are registered cases in every state of the country (MS 2016).

According to Luz et al. (2000), between 1930 and 1950, CL reached epidemic proportions to the north of Paraná during the colonisation period due to the replacement of native vegetation with coffee plantations. In the 1950s, the incidence of this zoonotic disease rapidly decreased due to the indirect effect of the malaria control campaigns about chlorinated insecticides (Luz et al. 2000). However, since 1980, CL has been endemic in Paraná (Lima et al. 2002). From 1980 to 2016, 16,464 cases were reported in the northern, western, and southwestern parts of the state (MS 2017).

In the state of Paraná, the natural life cycle of *Leishmania* is significantly associated with the natural forest areas that are close to the traditional agricultural production areas. The number of CL cases is highest in areas located in the basins of the Ivaí and Pirapó rivers, where there are native residual forests (Lima et al. 2002, Monteiro et al. 2008, Monteiro et al. 2009) as well as in the urban areas of Maringá (Teodoro et al. 1998, Lima et al. 2002) and Cianorte (Lima et al. 2002). Despite the substitution of native forest vegetation for coffee, soybean, corn, cotton, and pasture crops, the persistent incidence of CL in Paraná is the result of the successful adaptation of the vectors and reservoirs of parasites in the areas affected by human activities (Lima et al. 2002).

Data on the occupation of agrarian and urban spaces, vegetation cover, and the geographical distribution of CL allow the formulation of hypotheses and the planning of health services that control CL, particularly its vector (King et al. 2004, Monteiro et al. 2008). Therefore, the factors that influence the geographical distribution of CL were used in our statistical analysis using georeferencing, which will be extremely valuable in the planning of health initiatives (Medronho et al. 2003, Eisen and Eisen 2008, Magalhães 2012). The present study aimed at evaluating the association between CL and residual native vegetation, which is considered a risk factor for the prevalence of the disease, in the state of Paraná. Statistical analysis using georeferencing was carried out to assess this association.

## MATERIALS AND METHODS

*Description of the state of Paraná* - The state of Paraná is in southern Brazil (22º30’58” and 28º43’00” S; 48º05’37” and 54º37’08” W), with an area of 199,307.945 km^2^. It has 399 municipalities that are distributed across 10 macro-regions and 39 micro-regions. During the study period, the estimated population was 10,997,465, with a demographic density of 55.18 inhabitants/km and a human development index of 0.749 (IBGE 2010, IBGE 2016).

Paraná has three distinct climates according to the Köppen climate classification system. First, the tropical rainforest climate (megathermal, Af), which is restricted to the coastal strip, has an average temperature higher than 18ºC. Second, the humid subtropical climate (mesothermal, Cfa), which is the most common type of climate in the state, has an average temperature of 22ºC and can reach up to 40ºC in the North, Central-West, and the Ribeira River Valley regions during their warmest months. Meanwhile, its lowest average temperature is 18ºC. Finally, the temperature oceanic climate (mesothermal, Cfb) has an average temperature of 18-22ºC (IAPAR 2002).

*Data collection* - To analyse the influence of native vegetation on autochthonous cases of CL in the state of Paraná, data were obtained from the Sistema de Informação de Agravos de Notificação (SINAN; Information System for Notifiable Diseases) from January 2012 to December 2016. For the DC (autochthonous cases per 100,000 inhabitants), spatial density (SD) (cases per km^2^), altitude, and territorial area, the population and territorial data of each municipality were used based on the Instituto Brasileiro de Geografia e Estatística (Brazilian Institute for Geography and Statistics; IBGE 2010, IBGE 2016). Moreover, data on the residual native vegetation, river basins, and phytogeographical regions were provided by Fundação SOS Mata Atlântica (SOS Mata Atlântica Foundation) and Instituto Nacional de Pesquisas Espaciais (National Institute of Space Research, INPE; SOSMA 2017). Information on gender, age, clinical form, and the proportion of patients with CL who were cured was collected. In the present study, we focused on municipalities with SD values ≥ 0.010, which were considered as high-risk areas for disease transmission.

*Geographical and statistical analyses* - Geographical analysis was performed using the simultaneous autoregressive model (SAR) model (Bivand et al. 2008) that calculates the regression of values from several areas to model spatial dependence. Regression was based on the coefficient of the detection of CL (cases/100,000 inhabitants) as a function of the percentage of natural vegetation, altitude, the total number of cases, and SD (cases per km^2^). This result shows that the error is modeled based on its dependence on other errors. It is defined as

$$e_{i}\;=\;\sum_{i=1}^{m}\;b_{ij}e_{i}\;+\;{ɛ}_{i}\;,$$

where *b*_*ij*_ represents the values of spatial dependence between the areas and *ɛ*_*i*_ represents the residual error that is considered independent and identically distributed according to normal with zero mean and constant variance.

All statistical analyses were performed with R software (environment), with 95% confidence interval (CI). The collinearity between variables was evaluated, and the variables that were unstable were not used in the study (Development Core Team 2014).

## RESULTS

**Fig. 1:**
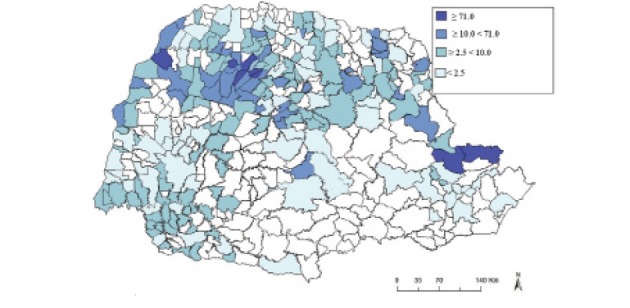
: distribution of cutaneous leishmaniosis cases in terms of detection coefficient (cases per 100,000 inhabitants) in the state of Paraná, Brazil, from 2012 to 2016.

From 2012 to 2016, there were 1600 (54.39%) reported cases of CL across 217 municipalities in the state of Paraná (Fig. 1, Table I). Among these, 8 (3.69%), 37 (17.05%), 102 (47.00%), and 70 (32.26%) municipalities had a DC of ≥ 71.0, ≥ 10.0 < 71.0, ≥ 2.5 < 10.0, and < 2.5, respectively. The following municipalities had the highest number of cases: Cianorte, Umuarama, Londrina, Jussara, Cerro Azul, and Maringá. Approximately 30.13% of CL cases were reported in these municipalities in 2012 and 2016. The following municipalities had the highest DC: Ivatuba, Jussara, São Jorge do Ivaí, Adrianópolis, Doutor Camargo, and Japurá (Table II), whereas 39 other municipalities had a DC ≥ 10.0, which include areas with high or extremely high CL transmission potential. Based on the analysis, municipalities, such as Ivatuba, Jussara, Japurá, Doutor Camargo, and Cianorte, had a significant SD (Table II).

**TABLE I:** Demographic and clinical characteristics of cutaneous leishmaniasis (CL) cases in the state of Paraná, Brazil, from 2012 to 2016 obtained using the chi-squared test

Characteristics of the participants	n	%	p-value
Sex			< 0.001
Male	1,187	74.19	
Female	413	25.81	
Age group (years)			< 0.001
> 1	10	0.63	
1-4	8	0.50	
5-9	32	2.00	
10-14	54	3.38	
15-19	86	5.38	
20-39	500	31.25	
40-59	561	35.06	
60-64	106	6.63	
65-69	102	6.38	
70-79	107	6.69	
≥ 80	34	2.13	
Clinical form			< 0.001
Cutaneous	1,445	90.31	
Mucocutaneous	154	9.61	
Not informed	1	0.06	
Case outcome			< 0.001
Clinical cure	1,029	64.31	
Abandonment of treatment	17	1.06	
Death related to CL	5	0.31	
Death from other causes	26	1.63	
Transfer	14	0.88	
Change of diagnosis	2	1.44	
Not informed	486	30.38	

**TABLE II:** Number, spatial density (SD), and detection coefficient (DC) of cutaneous leishmaniasis (CL) cases as well as natural vegetation covers in the municipalities with SD ≥ 0.010 in the state of Paraná, Brazil, from 2012 to 2016

Municipality	NC	SD	DC	V	Municipality	NC	SD	DC	V
Abatiá	6	0.026	15.43	2	Mandaguaçu	13	0.044	12.20	4
Adrianópolis	37	0.027	117.29	36	Mandaguari	5	0.015	2.95	7
Altônia	10	0.015	9.32	23	Maringá	59	0.121	3.02	3
Âmpere	4	0.013	4.37	4	Marmeleiro	7	0.018	9.78	6
Apucarana	14	0.025	2.18	10	Miraselva	2	0.022	21.37	7
Arapongas	10	0.026	1.76	8	Moreira Sales	4	0.011	6.33	2
Araruna	12	0.024	17.32	4	Nova Esperança	7	0.017	5.08	3
Bandeirantes	43	0.097	26.54	3	Nova Olímpia	2	0.015	7.00	4
Bela Vista do Paraíso	3	0.012	3.88	9	Paiçandu	10	0.058	5.17	2
Bom Sucesso	4	0.012	11.67	12	Paraíso do Norte	9	0.044	14.07	3
Cambé	13	0.026	2.54	5	Peabiru	7	0.015	10.00	6
Cambira	2	0.012	5.26	8	Perobal	4	0.010	13.52	3
Campo Mourão	29	0.038	6.33	8	Pinhais	3	0.049	0.48	0
Carlópolis	25	0.055	35.28	2	Pinhalão	10	0.045	31.44	6
Cerro Azul	64	0.048	72.94	6	Porecatu	3	0.010	4.30	6
Cianorte	131	0.161	34.39	9	Porto Rico	4	0.018	28.75	11
Corumbataí do Sul	2	0.012	10.58	6	Realeza	4	0.011	4.75	6
Cruzeiro do Sul	4	0.015	17.38	3	Rio Bom	4	0.022	23.96	6
Curitiba	9	0.021	0.09	1	Rolândia	5	0.011	1.61	4
Doutor Camargo	30	0.254	100.36	2	Sabaúdia	2	0.011	6.17	4
Engenehiro Beltrão	26	0.056	36.69	5	Salto do Lontra	3	0.010	4.18	4
Floresta	6	0.038	18.86	2	St. Amélia	2	0.026	10.84	6
Foz do Iguaçu	37	0.060	2.83	26	St. Cecília do Pavão	2	0.018	11.15	7
Francisco Beltrão	8	0.011	1.88	4	St. Izabel do Oeste	4	0.012	5.73	3
Grandes Rios	3	0.010	9.43	7	St. Terezinha do Itaipu	6	0.023	5.40	7
Guaíra	28	0.050	17.41	12	São Carlos do Ivaí	6	0.027	18.00	2
Ibaiti	10	0.011	6.61	7	São Jerônimo da Serra	21	0.025	36.65	12
Ibiporã	9	0.030	3.49	4	São Jorge do Ivaí	48	0.136	170.79	3
Icaraíma	39	0.058	90.32	4	São Jorge do Patrocínio	5	0.012	16.69	43
Indianópolis	2	0.016	9.03	3	São Manoel do Paraná	1	0.010	9.27	14
Itambaraca	12	0.058	35.28	2	São Tomé	19	0.087	68.04	12
Itaperuçu	6	0.019	4.58	10	Sarandi	8	0.077	1.80	2
Ivaiporã	7	0.016	4.32	3	Siqueira Campos	3	0.011	3.30	2
Ivatuba	40	0.414	253.32	1	Tapejara	10	0.017	12.93	5
Jandaia do Sul	3	0.016	2.86	9	Terra Boa	30	0.094	36.24	16
Japurá	43	0.260	95.18	5	Tomazina	23	0.039	53.43	4
Jardim Alegre	7	0.017	11.52	7	Tuneiras do Oeste	18	0.026	40.96	15
Jataizinho	4	0.025	6.48	5	Turvo	9	0.010	13.07	20
Jussara	73	0.346	212.27	10	Umuarama	80	0.065	15.00	6
Londrina	75	0.045	2.78	11	Uniflor	1	0.011	7.80	3
Lunardelli	3	0.015	11.75	14	Total	1356	0.042	28.81	

NC: number of cases; V: natural vegetation covers (%).

**Fig. 2:**
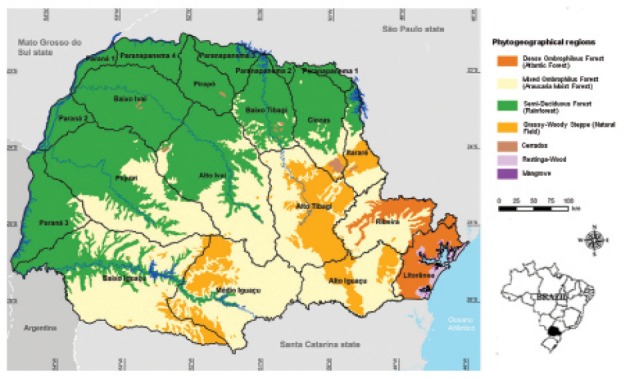
: phytogeographical regions and river basins in the state of Paraná, Brazil (Maack 1968).

**Fig. 3:**
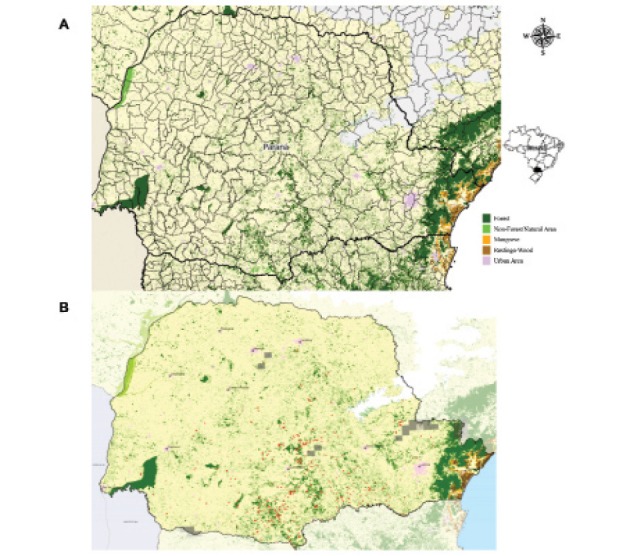
: residual vegetation cover in the state of Paraná from 2012 to 2013 (A) and from 2015 to 2016 (B) (SOSMA 2017).

Data on the native vegetation in the state of Paraná from 2012 to 2016 revealed that only 11.6% of the original vegetation cover remains (Figs 2-3). Spatial regression of DC revealed statistical significance only for SD (cases per km^2^) (Z = 24.1359, p < 0.05, 2012-2013; Z = 24.0817, p < 0.05, 2013-2014; Z = 33.4824, p < 0.05, 2014-2015; and Z = 27.1515, p < 0.05, 2015-2016) and the total number of cases (Z = 2.1146, p < 0.05, 2015-2016). Meanwhile, the values of other variables were as follows: for altitude, Z = -0.4840, p > 0.05, 2012-2013; Z = -0.9054, p > 0.05, 2013-2014; Z = -1.5980, p > 0.05, 2014-2015; Z = -1.6127, p > 0.05, 2015-2016; for the total number of cases, Z = -1.2357, p > 0.05, 2012-2013; Z = 1.1118, p > 0.05, 2013-2014; Z = 0.1938, p > 0.05, 2014-2015.

## DISCUSSION

Despite the significance of CL based on the expressive number of cases reported annually and its occurrence in all the states of Brazil, this disease continues to be neglected (Monteiro et al. 2008, MS 2016). Moreover, regardless of the increasing number of human interventions, the natural foci of CL are still found in rural and urban areas during the recent and past colonization (Lima et al. 2002, MS 2016).

Based on the regression analysis, a statistically significant difference (p < 0.05) was observed in terms of the SD of Ivatuba, Cianorte, Corumbataí do Sul, Doutor Camargo, Japurá, Jussara, Lunardelli, Mandaguaçu, Maringá, Moreira Sale, Paraíso do Norte, São Tomé, Terra Boa, Tuneiras do Oeste, and Umuarama, all of which are located in the CL generation hub, referred to as Ivaí-Pirapó hub by Monteiro et al. (2009). The semi-deciduous forest areas in these municipalities are represented by the fragments of natural vegetation cover that is moderately or severely altered (Lima et al*.* 2002, Monteiro et al. 2008, , Monteiro et al. 2009). No statistical significance (p > 0.05) was observed in terms of the vegetation factor per km^2^ and the percentage. Table II shows that Adrianópolis has 36% residual vegetation, with a high DC (117.29), whereas the municipality of Jussara has only 10%, with a high DC (212.27). Therefore, the life cycle of *Leishmania* is maintained regardless of the vegetation area in both the municipalities.

Although statistically significant difference was observed between the municipalities of Bandeirantes, Pinhalão, and Tomazina, which are included in the Cinzas-Laranjinha CL hub, in terms of SD data (p < 0.05) and natural vegetation cover (percentage and km^2^) (p > 0.05), these municipalities are located in the hydrography corridors of areas that are covered with semi-deciduous forest remnants, with conditions suitable for the transmission of *Leishmania* (Monteiro et al. 2009). The riparian forests are considered by the Brazilian Forest Code as a permanent preservation area, with several environmental functions and must respect an extension according to a river, lake, dam, or spring width. When damaged, the landowners must immediately restore the area. The situation is similar to that in the Tibagi hub, which includes the municipalities of Londrina and São Gerônimo da Serra, and a statistically significant difference was observed among these municipalities in term of SD data (p < 0.05) and natural vegetation cover factor (p > 0.05) (percentage).

In the Alto Ribeira hub, the municipalities of Adrianópolis and Cerro Azul, where the hydrography corridors exist, had statistically significant SD data (p < 0.05) and vegetation factor (p > 0.05) (per km^2^ and percentage). These corridors are covered by the Atlantic forest, which allows the coexistence of *Leishmania*, vectors, and reservoirs. The life cycle of *Leishmania* is maintained irrespective of the size of the natural vegetation that covers of the municipalities.

Although the municipalities with SD < 0.010 are not shown in Table II, all municipalities contribute in establishing the Paraná-Paranapanema and Ribeira circuits.

In the areas of the state of Paraná where the native vegetation was replaced by coffee crops in the 1930s and 1950s as well as by soy, maize, or pasture crops more recently (Lima et al. 2002, Monteiro et al. 2008, Monteiro et al. 2009), domestic and wild animal infection due to *Leishmania* spp. (Lonardoni et al. 2006, Membrive et al. 2012, Truppel et al. 2014), the collection of sandflies in the residual forest areas (Luz et al. 2000, Teodoro et al. 2001, Massafera et al. 2005, Cella et al. 2011, Cruz et al. 2013) and recreational fishing activities (Lima et al. 2002) partly explain the higher incidence of CL cases in these areas. These studies *in loco* provide significant details about CL epidemiology. According to Marlow et al. (2013), air humidity, temperature, rainfall, and other environmental and climatic variables may influence CL incidence over the time variables investigated at Santa Catarina. However, these variables cannot be identified using the methodology used in the present study.

The SINAN data used in the georeferencing provided a broad coverage of the population in 399 municipalities in the state, which facilitates the future continuity of the longitudinal study of CL epidemiology in Paraná, to assist with the health surveillance. The professionals who participated in the surveillance must be trained to improve the completeness of the SINAN data because errors were still observed during the completion of the notification forms. However, the georeferencing instruments must be cautiously used because the enforcement of variables alone and other computational models, without considering the local factors that affect the exposed population and their habitat, and the association between them, can cause technicians, managers, and researchers to obtain inaccurate results in case of multifactorial diseases (particularly CL) (Lovelock 2010). Therefore, other local factors, such as the influence of the work of individuals in agrarian and urban spaces, migratory behavior, and vegetation cover, that may influence the risk factors for CL occurrence must be investigated. These factors can determine spatial distribution, thus contributing to the occurrence of this disease in endemic areas (Monteiro et al. 2009).

*In conclusion* - Due to the significant association between vegetation areas and the ecology of vectors and reservoirs of CL, these areas have a significant influence on the prevalence of the disease. Thus, concentration of LT cases are commonly reported in the production hubs of this disease, which are areas with native vegetation, such as riparian forests. However, vegetation is not the only factor that influences the incidence of CL. Other factors, such as temperature, rainfall, and floating population, may also affect the prevalence of the disease.
